# Co‐infection of *Wolbachia* and *Spiroplasma* in spider mite *Tetranychus truncatus* increases male fitness

**DOI:** 10.1111/1744-7917.12696

**Published:** 2019-06-28

**Authors:** Kang Xie, Yi‐Jia Lu, Kun Yang, Shi‐Mei Huo, Xiao‐Yue Hong

**Affiliations:** ^1^ Department of Entomology Nanjing Agricultural University Nanjing China

**Keywords:** co‐infection, development time, *Spiroplasma*, *Tetranychus truncatus*, transcriptome, *Wolbachia*

## Abstract

*Wolbachia* and *Spiroplasma* are intracellular bacteria that are of great interest to entomologists, because of their ability to alter insect host biology in multiple ways. In the spider mite *Tetranychus truncatus*, co‐infection of *Wolbachia* and *Spiroplasma* can induce cytoplasmic incompatibility (CI) and fitness costs; however, little is known about the effect of co‐infection at the genetic level and the molecular mechanisms underlying CI. In this study, we explored the influence of the two symbionts on male mite host fitness and used RNA sequencing to generate the transcriptomes of *T. truncatus* with four different types of infection. In total, we found symbiont‐infected lines had a higher hatch proportion than the uninfected line, and the development time of the uninfected line was longer than that of the other lines. Co‐infection changed the expression of many genes related to digestion detoxification, reproduction, immunity and oxidation reduction. Our results indicate that co‐infection of *Wolbachia* and *Spiroplasma* confers multiple effects on their hosts, and helps illuminate the complex interactions between endosymbionts and arthropods.

## Introduction

*Wolbachia* is an intracellular bacterium which infects a large number of terrestrial arthropods (Zug & Hammerstein, 2012) and is famous for modulating host reproduction via induction of mechanisms that enhance its spread in insect host populations, including feminization, male killing, parthenogenesis and cytoplasmic incompatibility (CI) (Werren *et al*., 2008). Furthermore, *Wolbachia* also has positive effects on host fitness (Zug & Hammerstein, 2015). *Spiroplasma* is a Gram‐positive, wall‐less, helical bacteria that widely infects arthropods (Tully *et al*., 1983; Williamson *et al*., 1998; Gasparich *et al*., 2004). Some *Spiroplasma* strains are known to induce male killing in several insects, such as fruit flies, ladybird beetles, small brown planthoppers and butterflies (Williamson *et al*., 1999; Jiggins *et al*., 2000; Sanada *et al*., 2013; Tinsley & Majerus, 2006). In addition, many researchers found that *Spiroplasma* is a mutualistic symbiont that protects hosts against parasitoid wasps (Xie *et al*., 2010), nematodes (Jaenike *et al*., 2010; Jaenike & Brekke, 2011) and fungal pathogens (Scarborough *et al*., 2005).

Many studies have been performed to elucidate how symbiont infection influences host biology. A deubiquitylating enzyme encoded by *Wolbachia* was identified to induce CI (Beckmann *et al*., 2017). In *Drosophila melanogaster*, prophage WO genes *cifA* and *cifB* establish and enhance CI (LePage *et al*., 2017). In *Aedes aegypti* and *Anopheles gambiae*, it was observed that *Wolbachia* produced striking upregulation of many immune genes and inhibited pathogen development (Kambris *et al*., 2009; Kambris *et al*., 2010). In *D. melanogaster*, Harumoto and Lemaitre (2018) identified a protein from *Spiroplasma*, *Sp*AID, the expression of which induced male killing. In *Drosophila*, a ribosome‐inactivating protein encoded by *Spiroplasma* was proven to protect hosts against parasitic wasps and nematodes (Hamilton *et al*., 2016; Ballinger & Perlman, 2017). However, the biological effects of symbionts on hosts are always determined by host and/or symbiont genotype (Leonardo & Muiru, 2003), and it is necessary to study additional invertebrate systems to reveal the potential mechanisms of reproduction and fitness changes induced by endosymbiont co‐infection.

*Tetranychus truncatus* is a polyphagous agricultural pest that feeds on more than 60 plant species (Bolland *et al*., 1998) and has become a dominant pest in China in recent years (Zhang *et al*., 2018). Field investigation of *T. truncatus* has shown that co‐infection of *Wolbachia* and *Spiroplasma* was frequent in nature (Zhang *et al*., 2018). Our previous results showed that double infection of *Wolbachia* and *Spiroplasma* induced CI in *T. truncatus* (Zhang *et al*., 2018). A general explanation of CI is given by the “modification–rescue model”, in which one strain of *Wolbachia* both modifies sperm during spermatogenesis and “rescues” function to avoid embryonic development disruption (Werren, 1996; Hosokawa *et al*., 2010). In this model, males play the “modification” role. Although a previous study found that co‐infection could confer fitness benefits to their hosts as well as induce CI, little is known about the effect of co‐infection at the genetic level and the underlying molecular mechanisms of CI (Werren *et al*., 2008).

Here, we explored the effects of the two symbionts (*Wolbachia* and *Spiroplasma*) on male mite host fitness and compared gene expression profiles among four types of males from different symbiont‐infected lines, including doubly infected (Iws), singly *Wolbachia*‐infected (Iw), singly *Spiroplasma*‐infected (Is) and uninfected (U) lines. We found that symbiont‐infected lines had a higher hatch proportion than the uninfected line, and the development time of the uninfected line was longer relative to the other lines. Co‐infection changed the expression of many genes, including genes related to digestion detoxification, reproduction, immunity and oxidation reduction. Our results indicate that co‐infection of *Wolbachia* and *Spiroplasma* confers multiple effects on their hosts and helps elucidate the complex interactions between endosymbionts and arthropods.

## Materials and methods

### Mite rearing and sample collection

*Tetranychus truncatus* were collected from different sites in Shenyang, Liaoning Province, China. Mites were reared on leaves of kidney bean (*Phaseolus vulgaris* L.) at 25 ±1 °C, 60% relative humidity and under 16 : 8 L : D conditions. Three lines, Iws, Iw and Is, were established as previously described (Zhang *et al*., 2018). The U line was established by treating the Is line with tetracycline solution (0.1%, w/v) for three generations, and maintaining them in a mass‐rearing environment without antibiotic for seven generations before use to avoid potential side effects of antibiotic treatment.

To rule out the effects of host genetic background, females from the Iws, Iw and Is lines were allowed to mate with the males from the U line, and the female offspring continued to mate with males from the U line on new leaf disks. The above‐mentioned process was continued for seven generations until identical nuclear backgrounds were obtained. Then, spider mites were cultivated for five generations before experiments.

To ensure experiment accuracy, we checked the symbiont‐infected states using polymerase chain reaction (PCR).

### Hatchability assay

The effect of symbionts on male mite hatchability was assessed. For each line, a single 1‐day‐old adult virgin female was placed on a leaf disk and allowed to oviposit for 5 days. Each line used 36 leaf disks. To calculate hatch proportion, the eggs on the leaf disks were checked daily. Data were first tested for normality (Kolmogorov–Smirnov test) and homogeneity of group variances (Levene's test). When the data were normally distributed, they were analyzed with a one‐way analysis of variance with post‐hoc Tukey Honestly Significant Difference analysis for multiple comparisons. When the data did not follow a normal distribution, Kruskal–Wallis test with Bonferroni correction were used for multiple comparisons. All statistical analyses were done on SPSS 19.0 (IBM, Armonk, NY, USA).

### Development assay

To test whether symbiont infection influenced normal male development, we measured development time. For each line, approximately 150 3‐day‐old adult virgin females were placed on a leaf disk and allowed to oviposit for 3 h (3‐day‐old adult virgin females were in the active ovipositing state; it increased the possibility of obtaining more eggs in a relative short period). Then, the females were removed and eggs were left. The eggs on the leaf disks were checked at 8:00, 16:00, and 22:00 hours every day to determine whether males underwent eclosion. Observed adult males were picked out and the number of adult males recorded. Development time was considered the time which from an egg was laid to adulthood. The differences of development time were analyzed using the Kruskal–Wallis test with Bonferroni correction. All statistical analyses were done on SPSS 19.0.

### Library preparation and RNA sequencing

One‐day‐old adult virgin males of the four lines were then collected. Samples were stored in liquid nitrogen for subsequent RNA isolation.

Total RNA was extracted with the Trizol protocol according to manufacturer's instructions (Invitrogen, Carlsbad, CA, USA) for a total of 12 independent samples with three biological replicates per treatment. RNA integrity was assessed using the RNA Nano 6000 Assay Kit of the Agilent Bioanalyzer 2100 system (Agilent Technologies, Santa Clara, CA, USA). Libraries were generated using NEBNext® UltraTM RNA Library Prep Kit for Illumina® (NEB, Ipswich, MA, USA) following the manufacturer's recommendations, and index codes were added to attribute sequences to each sample. The complementary DNA (cDNA) library was then sequenced using Illumina Hiseq X Ten (Illumina, San Diego, CA, USA), and paired‐end reads were generated.

### Quality control

Raw data (raw reads) in fastq format were first processed through in‐house perl scripts. In this step, clean data (clean reads) were obtained by removing reads that contained adapters, reads that contained poly‐N, and low‐quality reads from raw data. Simultaneously, Q20, Q30, GC‐content and sequence duplication levels of the clean data were calculated. All downstream analyses were conducted with high‐quality clean data.

### Transcriptome assembly and gene functional annotation

Because there is no reference genome available for *T. truncatus*, a *de novo* transcriptome was built with Trinity v2.4.0 (Grabherr *et al*., 2011), with min_kmer_cov set to 2 and all other parameters set to default. Gene function was annotated based on the following databases: Nr (National Center for Biotechnology Information [NCBI] non‐redundant protein sequences); Nt (NCBI non‐redundant nucleotide sequences); Pfam (Protein family); EuKaryotic Orthologous Groups (KOG); Swiss‐Prot (a manually annotated and reviewed protein sequence database); KO (Kyoto Encyclopedia of Genes and Genomes [KEGG] Ortholog database); GO (Gene Ontology).

### Quantification of gene expression levels

Gene expression levels were estimated by RSEM (Li & Dewey, 2011) for each sample. Clean data were mapped back onto the assembled transcriptome, and read count for each gene was obtained from the mapping results.

### Differential expression analysis

Differential expression analysis of two conditions/groups was performed using the DESeq2 1.6.3 (Love *et al*., 2014). The *P*‐values were adjusted using the Benjamini and Hochberg (1995) method. Genes with an adjusted *P*‐value <0.05, as determined by DESeq2, were considered differentially expressed.

### Enrichment analysis of differentially expressed genes (DEGs)

GO enrichment analysis of the DEGs was implemented by the GOseq R packages based Wallenius non‐central hyper‐geometric distribution (Young *et al*., 2010), which can adjust for gene length bias in DEGs. KEGG (Kanehisa *et al*., 2007) is a database resource for understanding high‐level functions and utilities of a biological system, such as a cell, organism or ecosystem, from molecular‐level information, especially large‐scale molecular datasets generated by genome sequencing and other high‐throughput experimental technologies (http://www.genome.jp/kegg/). KOBAS (Mao *et al*., 2005) was used to test the statistical enrichment of DEGs in KEGG pathways.

### Quantitative real‐time PCR

To estimate the results of RNA sequence (RNA‐Seq) analysis, the expression levels of arbitrarily selected genes were measured by quantitative real‐time PCR (qPCR). The primer sequences are summarized in Table S1. Total RNA was extracted with the Trizol protocol. cDNA was synthesized according to the manufacturer's instructions (Takara, Kusatsu, Japan). qPCRs were performed on the Applied Bio‐systems 7500 Real‐Time PCR System with the qPCR SYBR Green Master Mix (Yeasen, China). Each sample was analyzed in triplicate in a 20‐*μ*L total reaction volume that contained 0.4 *μ*L (10 *μ*mol/L) of each primer, 7.2 *μ*L ddH_2_O, 10 *μ*L SYBR Green and 2 *μ*L diluted cDNA. The concentration of cDNA was too high for qPCR, so we diluted cDNA 5‐fold by adding ddH_2_O in order to get a viable cycle threshold (Ct) value. The relative expression levels were calculated using the 2^−ΔΔCt^ method (Livak & Schmittgen, 2001).

## Results

### Wolbachia and Spiroplasma have diverse effects on host hatchability

**Figure 1:**
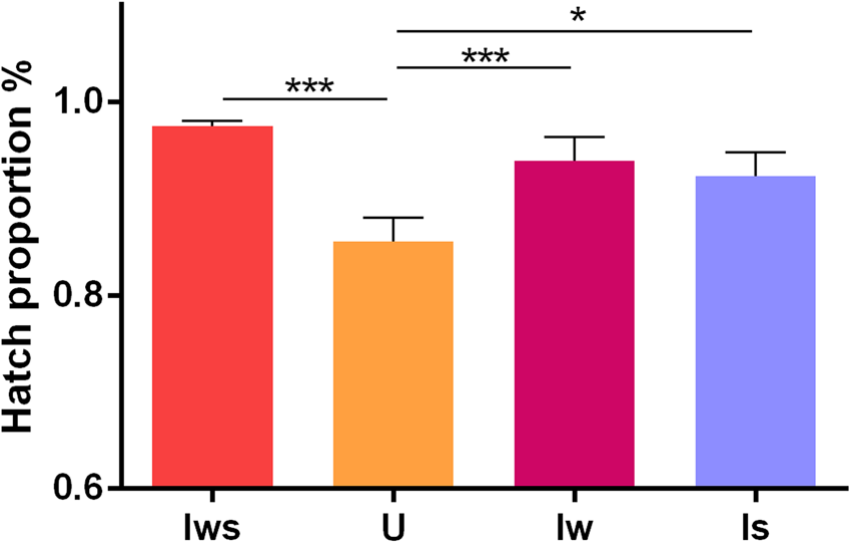
Hatch proportion of different male *Tetranychus truncatus* lines. Iws, doubly infected line; Iw, singly *Wolbachia*‐infected line; Is, singly *Spiroplasma*‐infected line; U, uninfected line. Results are mean ± SEM (Tukey Honestly Significant Difference test, *P* < 0.05).

The effects of *Wolbachia* and *Spiroplasma* on host hatchability were measured. As shown in Figure 1, the hatch proportion of uninfected males (0.8654 ± 0.0268) is significant lower than doubly infected males (Iws: 0.9747 ± 0.0052) (*P* < 0.001), singly *Wolbachia*‐infected males (Iw: 0.9384 ± 0.0249) (*P* < 0.001) and singly *Spiroplasma*‐infected males (Is: 0.9227 ± 0.0246) (*P* < 0.05). There was no significant difference between hatch proportions of symbiont‐infected males.

### Co‐infection shortened host developmental rate

**Figure 2:**
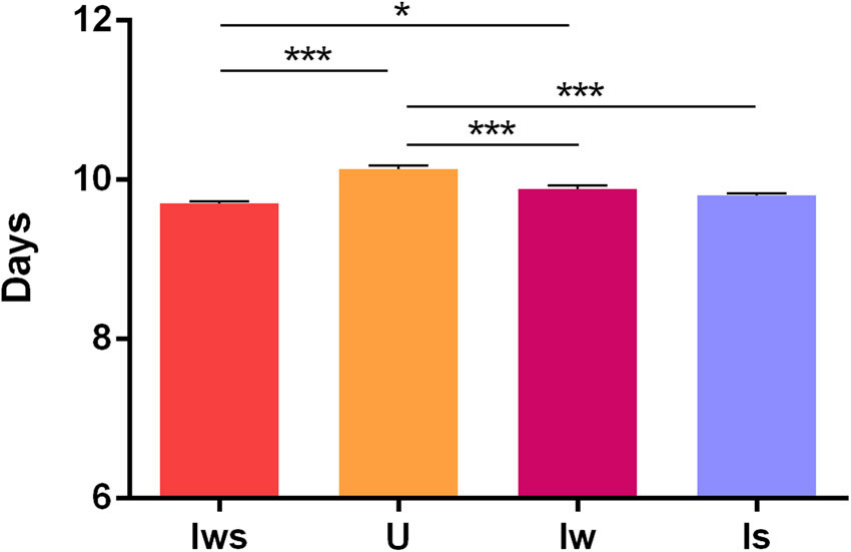
Development times of different male *Tetranychus truncatus* lines. Iws, doubly infected line; Iw, singly *Wolbachia*‐infected line; Is, singly *Spiroplasma*‐infected line; U, uninfected line. The differences of development time were analyzed using the Kruskal–Wallis test with Bonferroni correction (^*^ *P* < 0.05; ^***^ *P* < 0.001).

The development times of males from different lines are presented in Figure 2. Doubly infected males reached adulthood (9.71 ± 0.028) significantly earlier than singly *Wolbachia*‐infected males (9.89 ± 0.043) (*P* < 0.05). Additionally, uninfected males (10.13 ± 0.046) developed significantly slower than males of other lines (Is: 9.80 ± 0.034) (*P* < 0.001).

### RNA‐Seq data processing

**Figure 3:**
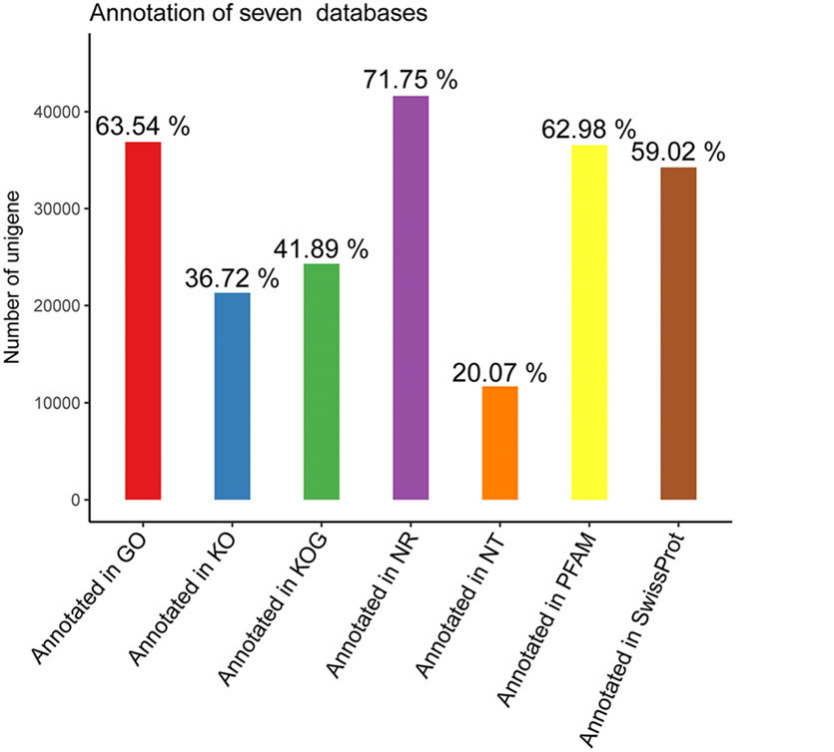
Number of unigenes annotated from each database.

**Figure 4:**
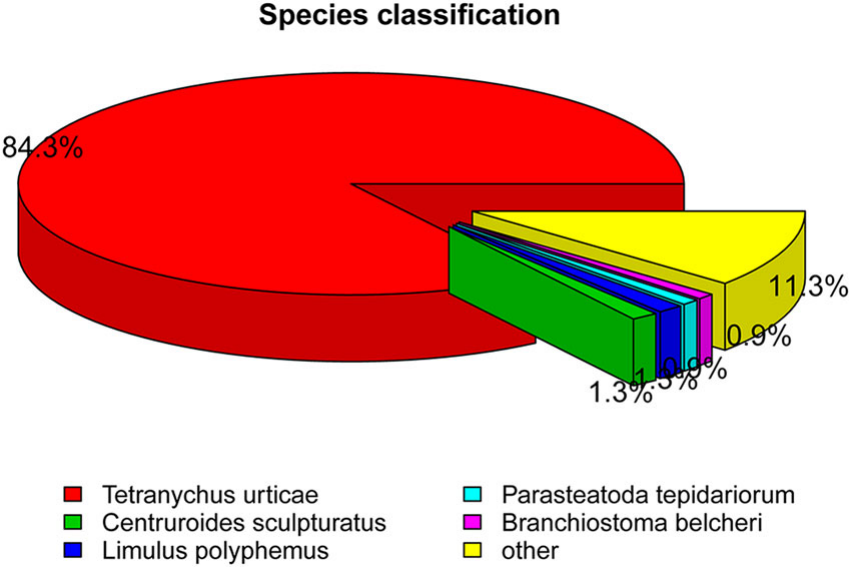
Species classification chart based on unigenes annotated from the Nr database; 84.3% of unigenes were assigned to *Tetranychus truncatus*.

Four transcriptome libraries (Iws, Iw, Is and U) were constructed, and each contained 50–54 million reads. A total of 58 015 unigenes were generated from 193 528 396 bp, with a median sequence length of 2408 bp and an N50 of 5468 bp. Over 32 000 unigenes were larger than 2 kb in size (Table 1). Because of the lack of a *T. truncatus* reference genome, all unigenes were annotated from the Nr (33827), Nt (11648), KO (21307), SwissProt (34170), PFAM (36542), GO (36868) and KOG (24304) databases (Fig 3). As shown in Figure 4, the vast majority of putative homologous genes were annotated to *T. urticae* by the Nr database, which was consistent with what we expected.

**Table 1:** Summary of sequencing results

			Sample name	
	Iws (%)	Iw (%)	Is (%)	U (%)
Raw reads	53428652	52031996	54450407	50926184
Clean reads	52453167	51200051	53717187	49889247
Total mapped	46903947 (89.42)	45414792 (88.70)	48057361 (89.46)	44151453 (88.50)
Total unigenes	58015			
Median length	2408			
N50	5456			
N90	1731			
Larger than 2 kb	32384			

Iws, doubly infected line; Iw, singly *Wolbachia*‐infected line; Is, singly *Spiroplasma*‐infected line; U, uninfected line.

**Figure 5:**
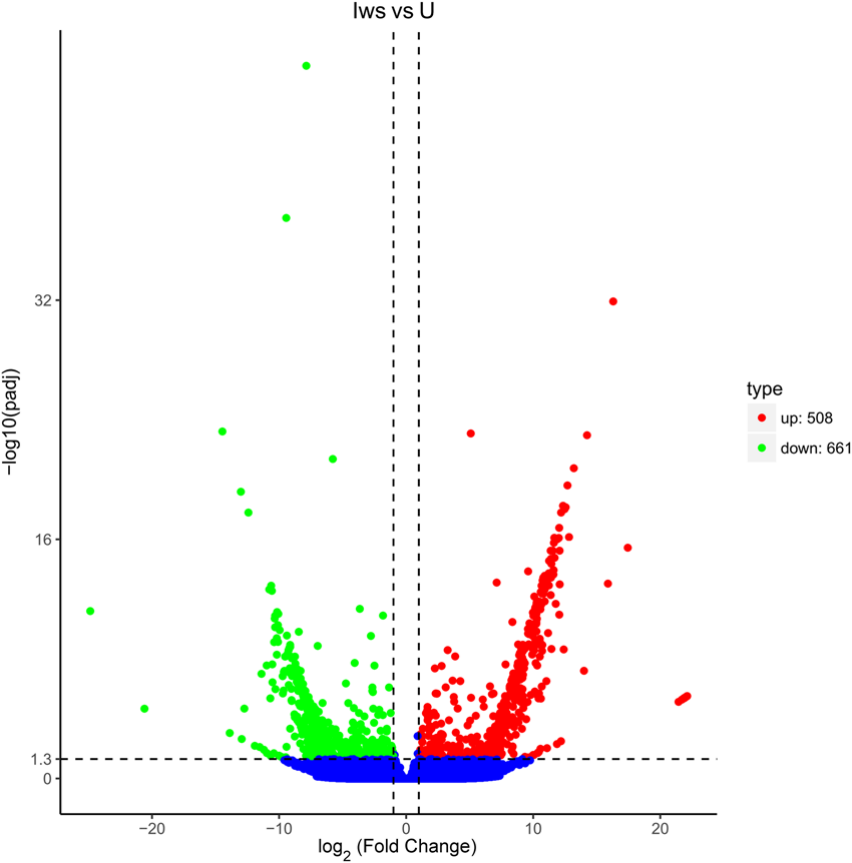
Volcano plot of differentially expressed genes in doubly infected (Iws) and uninfected (U) lines.

**Figure 6:**
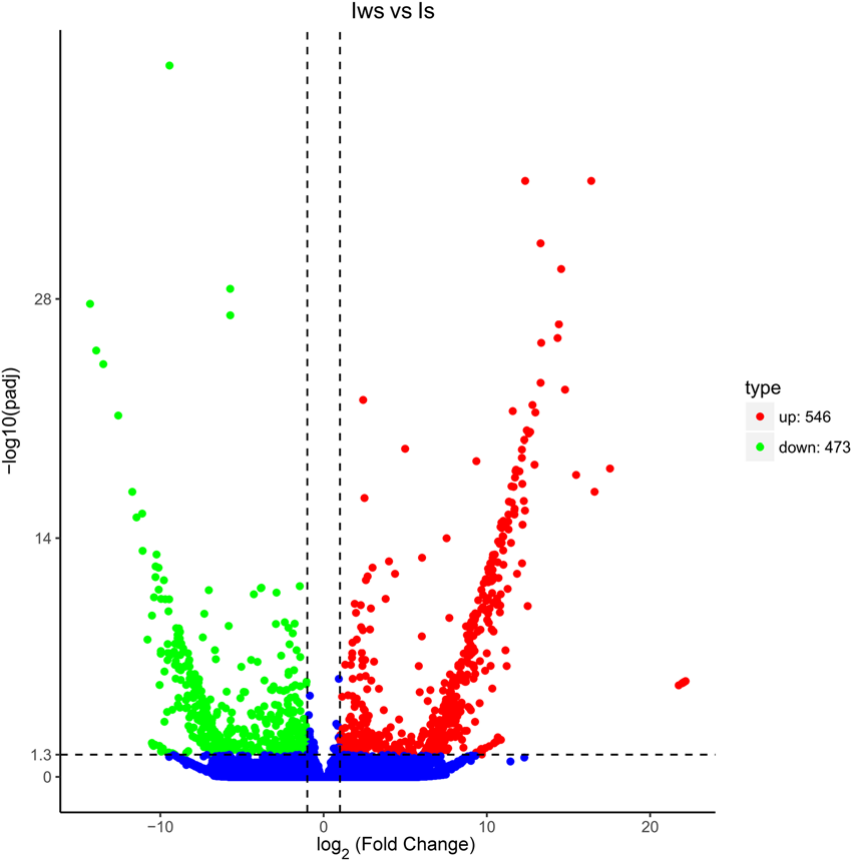
Volcano plot of differentially expressed genes in doubly infected (Iws) and singly *Spiroplasma*‐infected (Is) lines.

**Figure 7:**
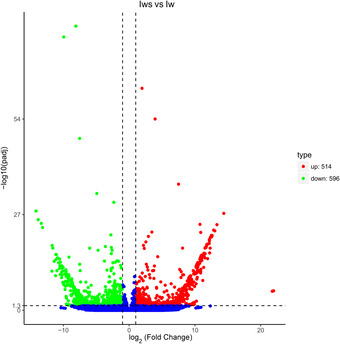
Volcano plot of differentially expressed genes in doubly infected (Iws) and singly *Wolbachia*‐infected (Iw) lines.

In total, 661 genes were downregulated and 508 were upregulated in Iws versus U (Fig. 5); 473 genes were downregulated and 546 were upregulated in Iws versus Is (Fig. 6); and 596 genes were downregulated and 514 were upregulated in Iws versus Iw (Fig. 7).

### GO analysis

**Figure 8:**
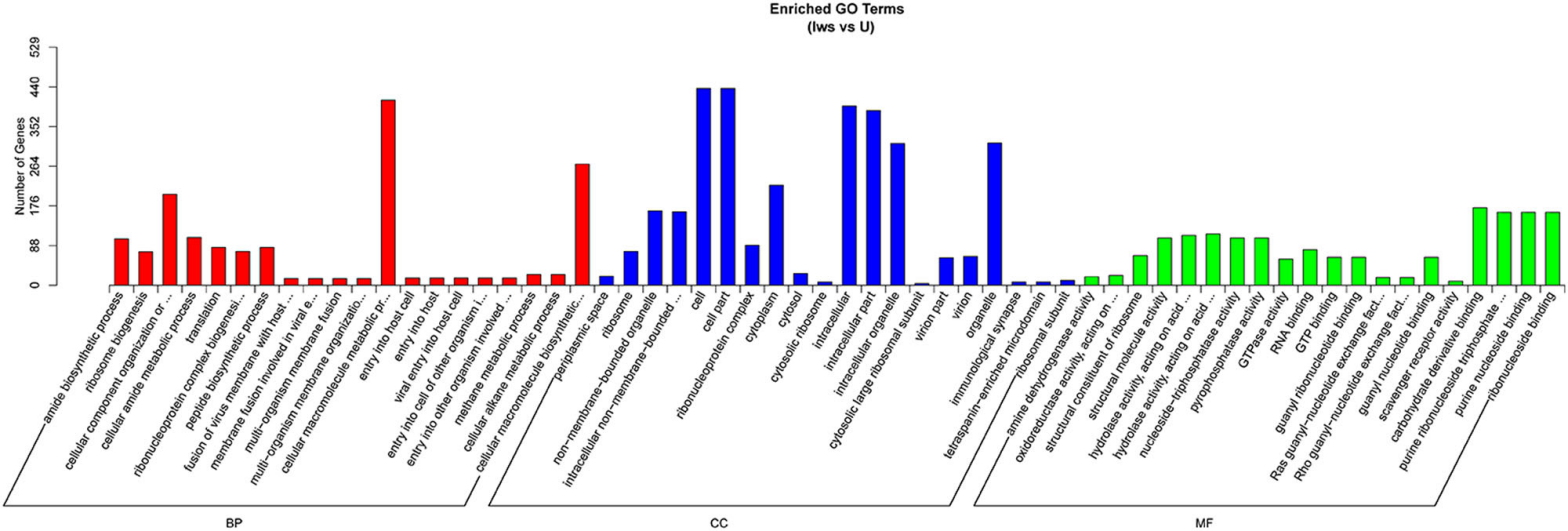
Gene Ontology enrichment analysis of differentially expressed genes between doubly infected (Iws) and uninfected (U) lines.

**Figure 9:**
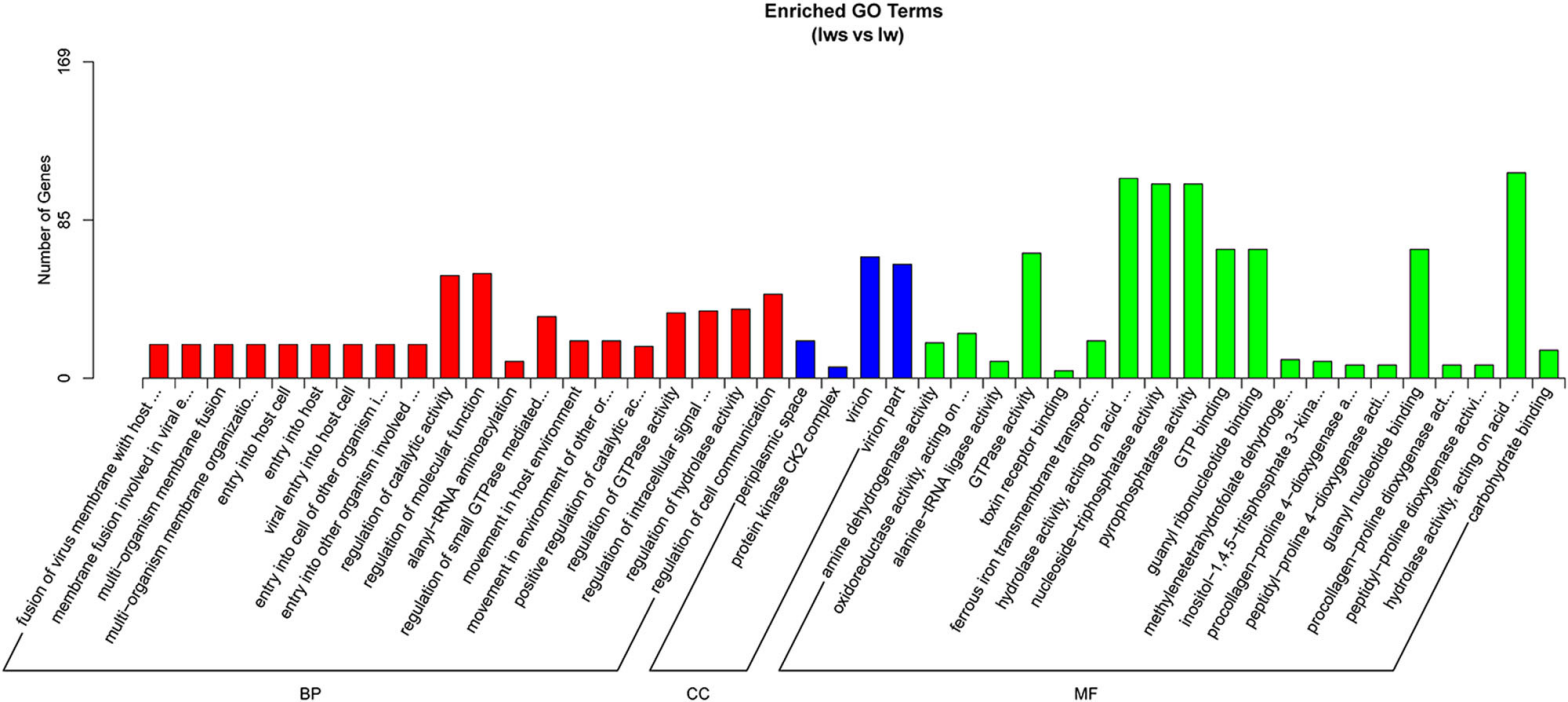
Gene Ontology enrichment analysis of differentially expressed genes between doubly infected (Iws) and singly *Wolbachia*‐infected (Iw) lines.

**Figure 10:**
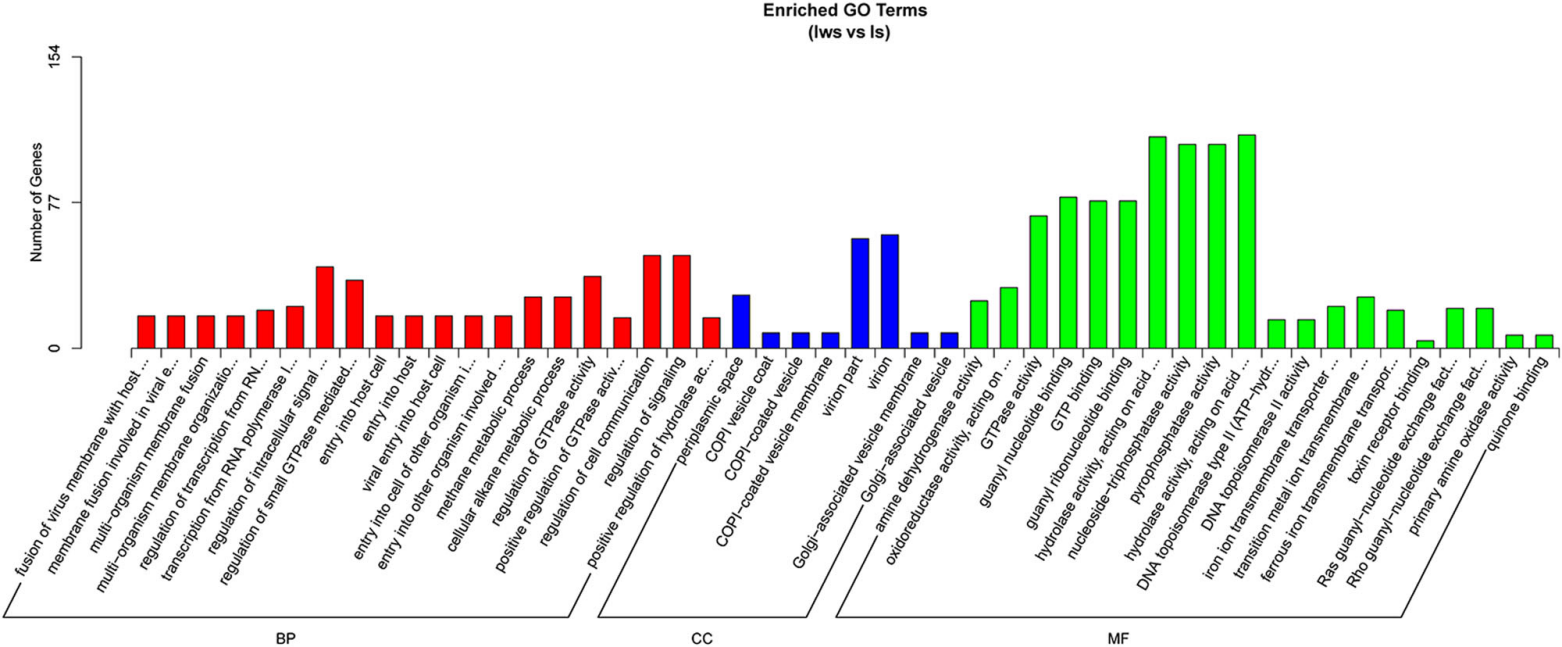
Gene Ontology enrichment analysis of differentially expressed genes between doubly infected (Iws) and singly *Spiroplasma*‐infected (Is) lines.

The gene functions of DEGs were annotated using the GO database, and here we list terms which were the most significant enrichment of DEGs in Iws compared with the other three lines. In Iws versus U, DEGs were most enriched in the biological process (BP) categories of amide biosynthetic process and ribosome biogenesis (Fig. 8). In Iws versus Is and Iws versus Iw, fusion of virus membrane with host plasma membrane and membrane fusion involved in viral entry into host cells were the most significant enrichment BP terms. Periplasmic space was the strongest enriched term in the cellular component category in all three comparisons. Additionally, amine dehydrogenase activity and oxidoreductase activity, acting on the CH‐NH2 group of donors were the most significant enrichment terms associated with the molecular function category in all three comparisons (Figs. 8, 9, 10).

### KEGG pathway analysis

**Figure 11:**
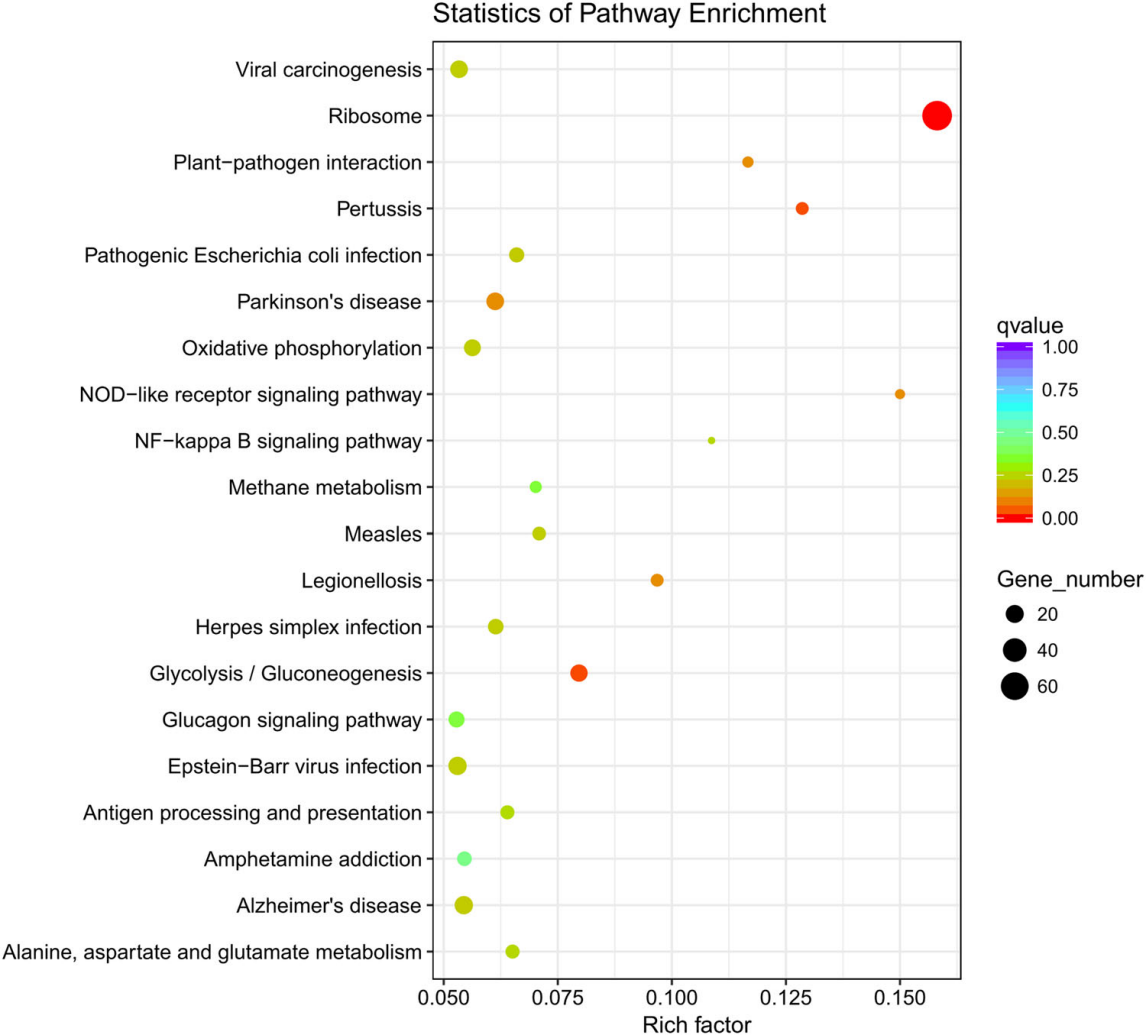
The 20 most enriched Kyoto Encyclopedia of Genes and Genomes (KEGG) pathways based on differentially expressed genes between doubly infected (Iws) and uninfected (U) lines. The *x*‐axis shows the rich factor. The *y*‐axis shows the pathway name. The point size represents the number of genes enriched in a particular pathway. Degree of enrichment is more significant for larger rich factor values and smaller q values.

**Figure 12:**
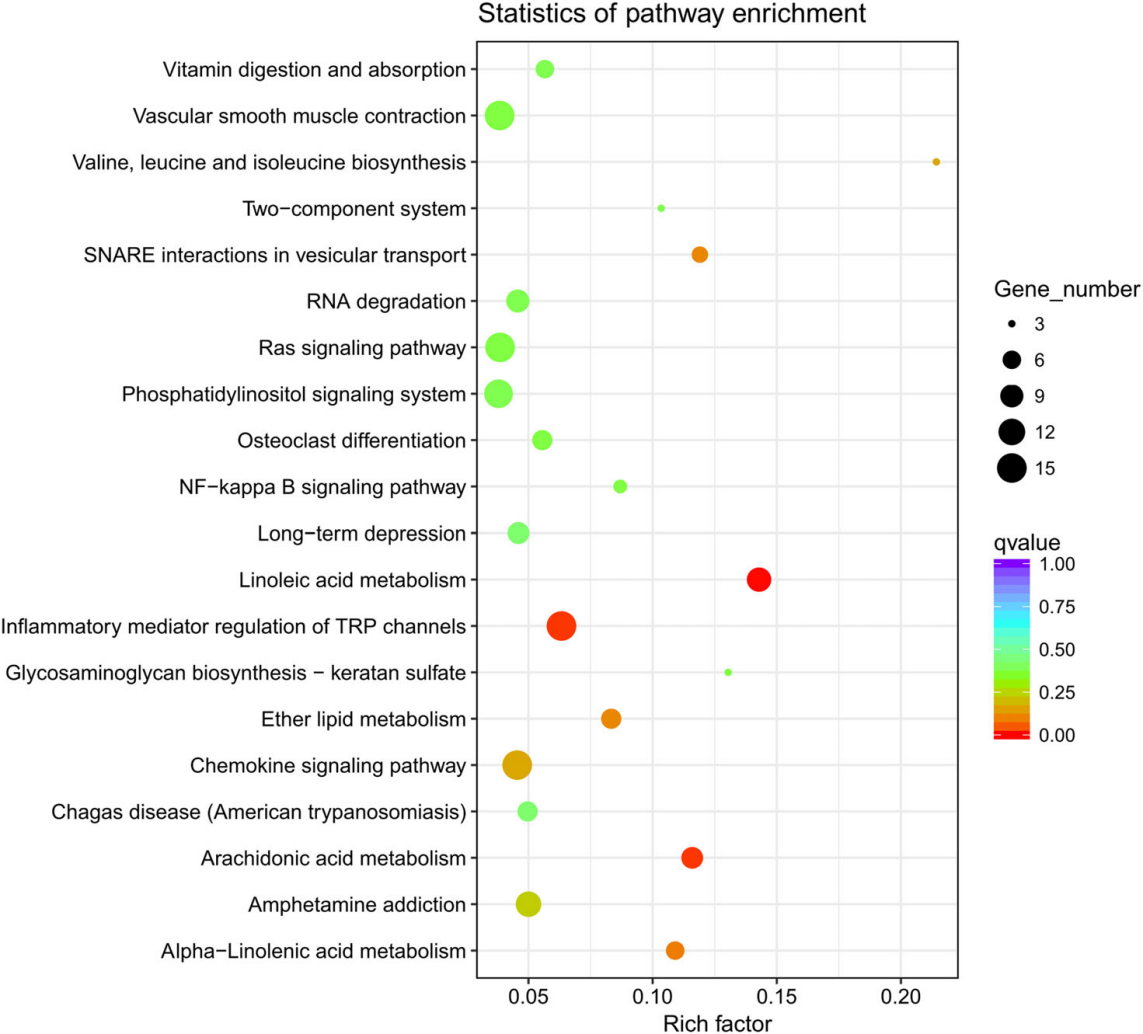
The 20 most enriched Kyoto Encyclopedia of Genes and Genomes (KEGG) pathways based on differentially expressed genes between doubly infected (Iws) and singly *Spiroplasma*‐infected (Is) lines. The *x*‐axis shows the rich factor. The *y*‐axis shows the pathway name. The point size represents the number of genes enriched in a particular pathway. Degree of enrichment is more significant for larger rich factor values and smaller q values.

**Figure 13:**
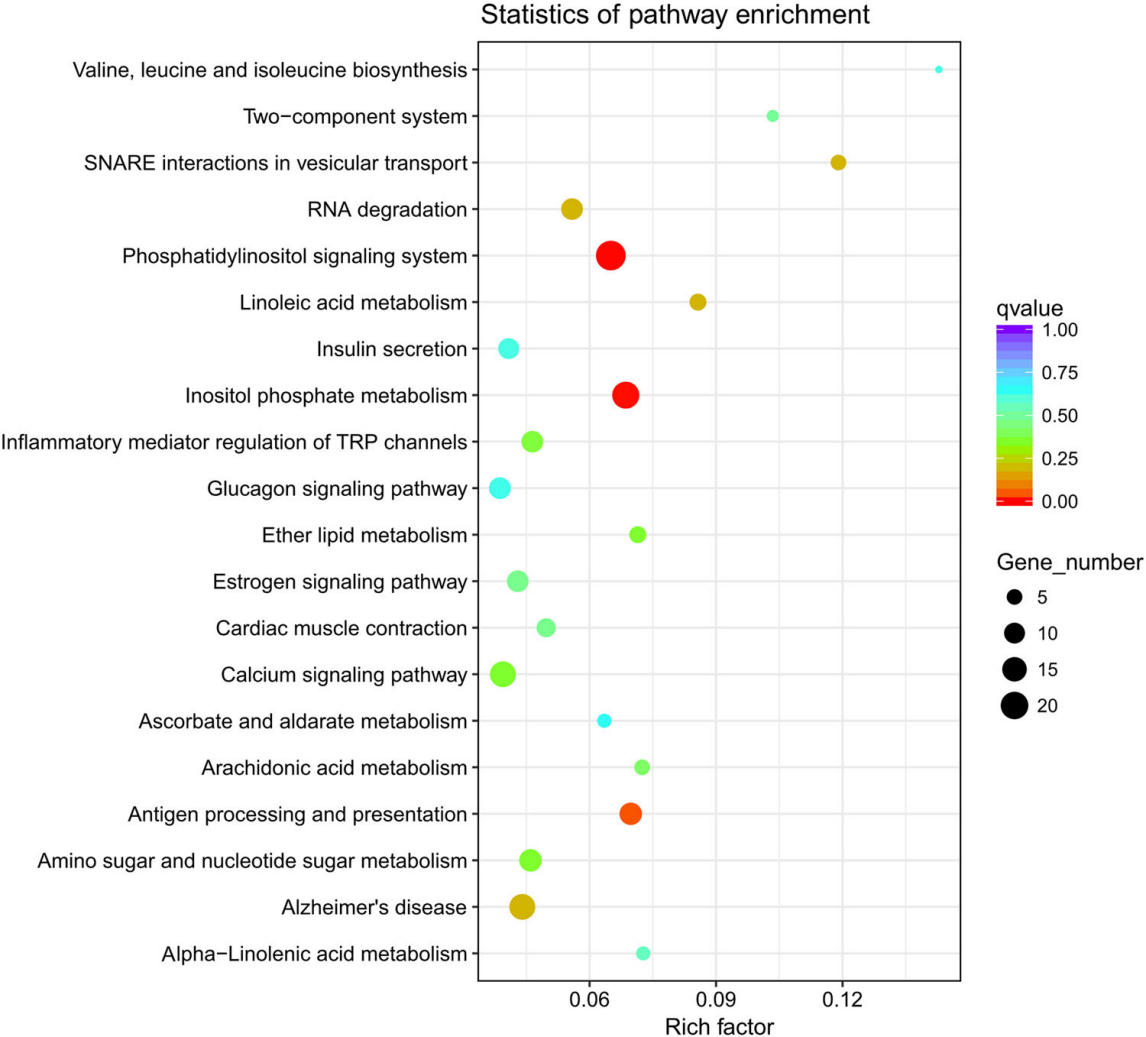
The 20 most enriched Kyoto Encyclopedia of Genes and Genomes (KEGG) pathways based on differentially expressed genes between doubly infected (Iws) and singly *Wolbachia*‐infected (Iw) lines. The *x*‐axis shows the rich factor. The *y*‐axis shows the pathway name. The point size represents the number of genes enriched in a particular pathway. Degree of enrichment is more significant for larger rich factor values and smaller q values.

In Iws versus U, KEGG pathway analysis showed that DEGs were involved in 277 different pathways, mainly including those related to ribosomes and glycolysis/gluconeogenesis (Fig. 11). In Iws versus Is, DEGs were involved in 260 different pathways, mainly including linoleic acid metabolism and arachidonic acid metabolism (Fig. 12). In Iws versus Iw, genes were involved in 263 different pathways, mainly including those related to the phosphatidylinositol signaling system and inositol phosphate metabolism (Fig. 13).

### DEGs of interest

Co‐infection of *Wolbachia* and *Spiroplasma* changed the expression of many more genes compared with other types of symbiont infection (Table 2).

**Table 2:** Subset of identified differentially expressed genes

			log_2_ (Fold Change)		
Gene category	Gene ID	Description	Iws vs U	Iws vs Is	Iws vs Iw
Digestion or detoxification	*Cluster‐6764.35608*	Acetylcholinesterase‐like precursor	−3.9453	−3.8502	−3.5378
	*Cluster‐6764.37341*	Acetylcholinesterase‐like precursor	−9.4156	–	–
	*Cluster‐6764.37244*	Acetylcholinesterase‐1	–	−6.594	–
	*Cluster‐6764.39802*	Cholinesterase	11.407	14.79	–
	*Cluster‐6764.39914*	Cholinesterase	11.212	11.31	–
	*Cluster‐6764.39792*	Cholinesterase	7.1257	7.5336	–
	*Cluster‐6764.39797*	Cholinesterase	8.309	6.5407	–
	*Cluster‐6764.39798*	Cholinesterase	7.3991	7.4976	–
	*Cluster‐6764.4172*	Cholinesterase	7.1807	7.2838	–
	*Cluster‐6764.39800*	Cholinesterase	16.297	16.394	–
	*Cluster‐6764.23573*	Multidrug resistance‐associated protein 1	22.075	22.171	–
	*Cluster‐6764.6902*	Multidrug resistance‐associated protein 1	−6.7113	–	−6.0865
	*Cluster‐6764.32781*	Multidrug resistance‐associated protein 4	8.8705	–	–
	*Cluster‐6764.19713*	Multidrug resistance‐associated protein 1	–	−3.8554	–
	*Cluster‐6764.25271*	Multidrug resistance‐associated protein 1	–	−6.3218	–
	*Cluster‐6764.18981*	Multidrug resistance‐associated protein 1	–	4.4568	–
	*Cluster‐6764.34008*	Multidrug resistance‐associated protein 1	–	3.3699	2.3407
	*Cluster‐6764.5965*	Multidrug resistance‐associated protein 4	–	−1.6863	–
	*Cluster‐6764.19020*	Multidrug resistance‐associated protein 1	–	–	−6.7381
	*Cluster‐6764.8855*	Multidrug resistance‐associated protein 4	–	–	4.4642
	*Cluster‐6764.4892*	Multidrug resistance‐associated protein 4	–	–	1.9779
	*Cluster‐6764.13758*	Canalicular multispecific organic anion transporter 1	–	–	−4.7031
	*Cluster‐6764.22981*	Canalicular multispecific organic anion transporter 2	–	–	−1.2515
	*Cluster‐6764.8292*	UDP‐glucosyltransferase YdhE	−5.8003	–	–
	*Cluster‐6764.28542*	UDP‐glucosyltransferase YjiC	8.2717	5.8864	–
	*Cluster‐6764.31354*	UDP‐glucosyltransferase YjiC	−6.7652	–	–
	*Cluster‐6764.17582*	UDP‐glucosyltransferase YjiC	6.852	–	–
	*Cluster‐6764.17581*	UDP‐glucosyltransferase YjiC	–	1.6587	–
	*Cluster‐6764.37988*	UDP‐glucosyltransferase YjiC	–	−6.3608	–
	*Cluster‐6764.28477*	UDP‐glucose:glycoprotein glucosyltransferase 1	–	–	10.187
	*Cluster‐6764.10305*	UDP‐glucosyltransferase YjiC	–	–	−1.7827
	*Cluster‐6764.37989*	UDP‐glycosyltransferases	–	–	−2.5221
	*Cluster‐6764.19594*	UDP‐glucosyltransferase YojK	–	–	−4.7457
Lipocalins	*Cluster‐6764.39035*	Apolipoprotein D	−3.7197	−4.2719	–
	*Cluster‐6764.21793*	Apolipoprotein D	−1.4002	–	–
	*Cluster‐6764.7749*	Apolipoprotein D	−7.2849	–	–
	*Cluster‐6764.20038*	Apolipoprotein D	–	−9.2612	−9.1475
	*Cluster‐6764.30554*	Apolipoprotein D	–	–	5.8735
	*Cluster‐6764.4653*	Apolipoprotein D	–	–	−6.3934
Reproduction	*Cluster‐1760.0*	Histone variant H2A.Z	−6.1468	–	–
	*Cluster‐6764.36412*	Histone deacetylase complex subunit SAP130	1.1359	–	–
	*Cluster‐11188.0*	Histone H3.3	−7.39	–	–
	*Cluster‐6764.8724*	Histone‐lysine N‐methyltransferase SETD1B	–	−7.3224	–
	*Cluster‐6764.43656*	Histone‐lysine N‐methyltransferase SETMAR	–	−3.06	−3.3232
	*Cluster‐6764.31921*	Histone‐lysine N‐methyltransferase SETD1B	–	–	2.5072
	*Cluster‐6764.12902*	Integrin beta pat‐3	–	–	−1.1589
	*Cluster‐6764.12904*	Integrin beta pat‐3	–	–	1.0747
	*Cluster‐6764.23516*	Cyclin‐G‐associated kinase	2.1506	–	–
	*Cluster‐6764.24423*	DNA topoisomerase 2‐alpha	14.238	14.334	14.391
	*Cluster‐6764.19985*	DNA topoisomerase 2‐alpha	13.197	13.294	13.351
	*Cluster‐6764.22770*	DNA topoisomerase 2‐alpha	12.187	–	–
	*Cluster‐6764.16349*	DNA topoisomerase 2‐alpha	–	−1.9319	−2.0822
	*Cluster‐6764.21733*	DNA topoisomerase 2‐alpha	–	−1.7772	−2.1526
	*Cluster‐6764.23918*	Ecdysone‐inducible protein E75	–	–	1.8406
	*Cluster‐6764.31632*	Ecdysone‐induced protein 78C	–	1.1858	1.2414
Immunity	*Cluster‐6764.16214*	Serine/threonine‐protein kinase pelle	9.4517	9.5598	–
	*Cluster‐6764.24389*	Nuclear factor‐kappa‐B p100 subunit	11.588	11.688	–
	*Cluster‐6764.34859*	Nuclear factor‐kappa‐B p105 subunit	7.7836	–	–
	*Cluster‐6764.24390*	Nuclear factor‐kappa‐B p100 subunit	11.605	11.701	–
	*Cluster‐6764.10026*	Nuclear factor‐kappa‐B p105 subunit	−8.0926	−8.0389	−8.5413
	*Cluster‐6764.11921*	JAK/STAT pathway ortholog of Dm stat92E	4.5353	–	–
	*Cluster‐6764.11909*	JAK/STAT pathway ortholog of Dm stat92E	4.8424	–	–
	*Cluster‐6764.11905*	JAK/STAT pathway ortholog of Dm stat92E	5.5734	–	–
	*Cluster‐6764.32494*	Toll‐like receptor Tollo	–	1.2988	–
Oxidation reduction process	*Cluster‐6764.39632*	Dual oxidase 2	–	–	6.662
	*Cluster‐6764.32305*	Dual oxidase maturation factor 2	–	–	9.3804
	*Cluster‐6764.32307*	Dual oxidase maturation factor 2	–	–	7.7968
	*Cluster‐4417.0*	Peroxidase	−5.8252	–	–
	*Cluster‐6764.38953*	Peroxidase	–	–	2.0365
	*Cluster‐6764.25509*	Short‐chain dehydrogenase	–	−9.4161	−9.4985
	*Cluster‐6764.25508*	Short‐chain dehydrogenase	–	−8.7428	−8.4418
	*Cluster‐599.1*	Ferritin, heavy subunit	−7.1255	–	–
	*Cluster‐8341.0*	Ferritin, middle subunit	−7.3421	–	–
Structural constituent of cuticle	*Cluster‐6764.21118*	Adult‐specific rigid cuticular protein 15.7	−2.7772	−2.5967	−2.3873
	*Cluster‐6764.22958*	Adult‐specific rigid cuticular protein 15.7	−2.1123	−2.326	−2.1123

Iws, doubly infected line; Iw, singly *Wolbachia*‐infected line; Is, singly *Spiroplasma*‐infected line; U, uninfected line.

The polyphagy of *T. truncatus* could be attributed to powerful detoxification functions, such as having a series of detoxifying enzymes, including cytochrome P450 monooxygenases (CYPs), glutathione *S*‐transferase, ABC transporter, carboxylesterase and multidrug resistance‐associated protein. Co‐infection of the two symbionts had different degrees of influence on the expression of these genes in a host. For CYP genes, six genes significantly changed in Iws, including four downregulated and two upregulated genes. Four genes that encode glutathione‐*S*‐transferase were downregulated (three genes were downregulated compared with Is, and one gene was downregulated compared with U). Additionally, for two genes that encode ABC transporters, one gene was upregulated and one gene was downregulated. Moreover, one carboxylesterase gene was downregulated. Many genes that encode multidrug resistance‐associated protein dramatically changed, including five downregulated and six upregulated genes. Several other genes were also downregulated, including those that encode phosphatase, canalicular multispecific organic anion transporter and P‐glycoprotein.

Lipocalins are a family of proteins which transport small hydrophobic molecules. We found that the majority of genes which encode apolipoprotein D were downregulated and only one was upregulated. Several genes associated with larval molt were upregulated. There were several genes related to histone coding and histone modification (*Cluster‐1760.0*, *Cluster‐6764.36412*, *Cluster‐11188.0*, *Cluster‐6764.8724*, *Cluster‐6764.43656*); four of five of these genes were downregulated. We also observed downregulation of three genes related to vitellogenin. Two genes, *Cluster‐6764.21118* and *Cluster‐6764.22958*, which participate in cuticle biosynthesis, were downregulated. Moreover, it is noteworthy that the genes that encode DNA topoisomerase 2‐alpha, which are essential for proper segregation of daughter chromosomes during mitosis and meiosis, were all substantially upregulated.

### qPCR validation

**Figure 14:**
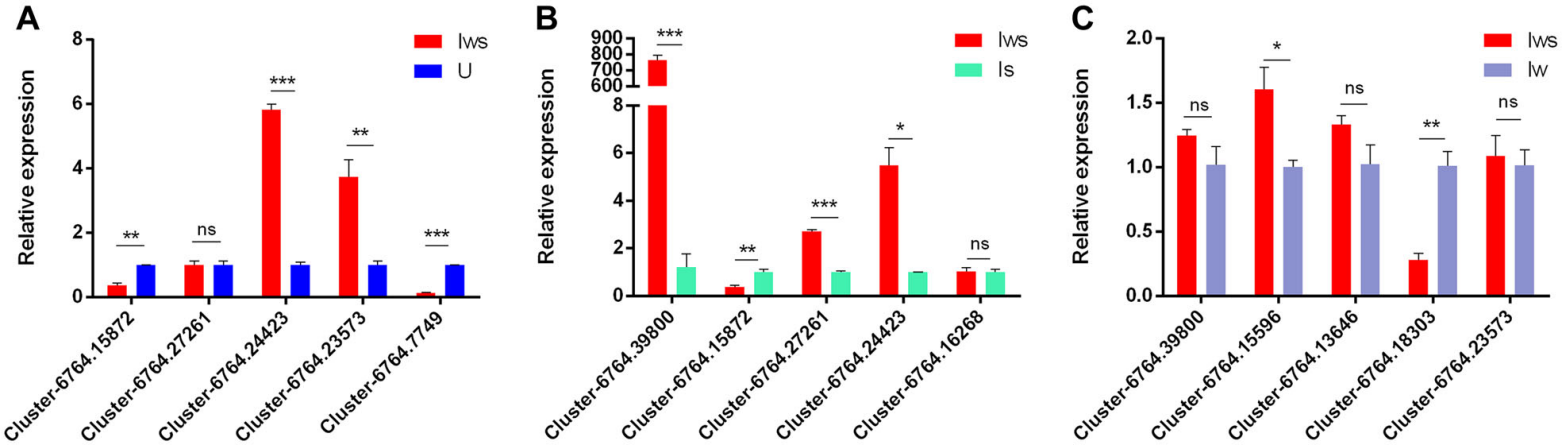
Expression patterns of differentially expressed genes in different symbiont‐infected lines. (A) Doubly infected (Iws) compared with uninfected (U) lines. (B) Iws compared with singly *Spiroplasma*‐infected (Is) lines. (C) Iws compared with singly *Wolbachia*‐infected (Iw) lines. Relative expression was calculated using the 2^−ΔΔCt^ method. Data are presented as mean ± SEM.

Ten DEGs were arbitrarily picked to confirm sequencing data accuracy (Fig. 14). We examined the expression of these genes in an arbitrary comparison. The differences in expression of all genes except *Cluster‐6764.13646* were consistent with RNA‐Seq results. These results support the reliability of the RNA‐Seq data.

## Discussion

It is common in nature for multiple endosymbionts to infect the same host (Duron *et al*., 2008). Co‐infection with *Wolbachia* and *Spiroplasma* could have various effects on host reproduction and fitness. Because the effects of symbionts on host biology differ among host and/or symbiont genotypes (Serbus *et al*., 2008), it is necessary to study additional invertebrate systems to reveal the potential mechanisms of reproduction and fitness changes induced by symbiont co‐infection. In addition, a common CI defect is a delay in the breakdown of the male nuclear envelope, which results in paternal chromosome mis‐segregation during the first mitosis (Tram & Sullivan, 2002; Tram *et al*., 2006; Gebiola *et al*., 2017). Additionally, increased male age can dramatically decrease the strength of CI (Kittayapong *et al*., 2002; Reynolds & Hoffmann, 2002). Therefore, we collected 1‐day‐old adult virgin males for the transcriptome analysis in this study.

*T. truncatus* is a polyphagous herbivore and has numerous genes related to detoxification. In our study, the expression of these specialized genes dramatically changed as a result of symbiont co‐infection, which indicates that co‐infection of *Wolbachia* and *Spiroplasma* affects *T. truncatus* feeding and digestion. We observed that 34 genes related to digestion and detoxification were differentially expressed in the Iws line, which indicates that the co‐infection largely changed host detoxification ability. There were also remarkable changes in many genes which encode canalicular multispecific organic anion transporter and multidrug resistance‐associated proteins. They are members of the ABCC subfamily. ABCC proteins have a series of functions, including ion transport, cell‐surface receptor activity and translocating many kinds of substrates, such as drugs, cyclic nucleotides, endogenous compounds and their glutathione conjugates and glutathione (Dermauw *et al*., 2013). The majority of genes that encode cholinesterase were upregulated in Iws. Cholinesterase plays an important role in hydrolyzing both plant secondary metabolites, organophosphates and other pesticides (Ramsey *et al*., 2010). Many lipocalin genes were differentially expressed. Lipocalins are a family of proteins capable of binding to small hydrophobic molecules (Flower, 1996). In spider mites, they are likely to bind pesticides/allelochemicals, which results in sequestration of these toxic, generally hydrophobic compounds. Furthermore, we found that UDP‐glycosyltransferases (UGTs), which catalyze the addition of the glycosyl group from a UTP‐sugar to a small hydrophobic molecule, were strongly differentially expressed in Iws. One of the principle functions of insect UGTs is detoxification of ingested plant phenolics, which act as toxins, or insect deterrents (Ahmad & Hopkins, 1993).

In fact, for herbivores, the nutrient content provided by plant material is usually poor or unbalanced (Karban & Baldwin, 2007). The detoxification ability of herbivores enhances utilization of limited plant nutrition. In spider mites, nutritional status is related to host performance (Wermelinger *et al*., 1991). Our previous study showed that co‐infection of *Wolbachia* and *Spiroplasma* could confer some fitness benefits to *T. truncatus* hosts, including shortened development time, and increased fecundity and hatchability, which can all contribute to the expansion of symbionts in host populations (Zhang *et al*., 2018). Hence, the detoxification ability of *T. truncatus* has an indirect impact on the spread of symbionts. We conclude that the strong detoxification ability of the doubly infected line was at least one reason why co‐infection of *Wolbachia* and *Spiroplasma* is widespread in natural *T. truncatus* populations (Zhao *et al*., 2013; Zhang *et al*., 2016). However, the entirely different symbiont prevalence in the field (Zhu *et al*., 2018) probably reflects different levels of detoxification ability of various mite lines. For example, *Wolbachia* are largely present in the gnathostoma of spider mites (Zhao *et al*., 2013), and the function of *Wolbachia* is related to their localization, which favors the perspective that symbiont infection affects food digestion and detoxification. Further studies are needed to confirm the influence of symbionts on detoxification.

Some genes related to reproduction were differentially expressed as a result of co‐infection. Two genes that encode a cuticle protein were downregulated in Iws. In the nematode *Brugia malayi*, *Wolbachia* removal resulted in downregulated transcripts involved in cuticle biosynthesis, which may reflect disruption of a normal embryogenic process (Ghedin *et al*., 2009). Moreover, two genes that encode integrin beta pat‐3 were differentially expressed. During *Caenorhabditis elegans* development, pat‐3 is essential for gonad morphogenesis, and *pat‐3* RNAi caused abnormal gonad formation and sterility (Lee *et al*., 2001; Lee *et al*., 2005; Xu *et al*., 2005). Substantial changes were observed in several genes associated with histone modifications and histone variants. These genes were found to participate in various cell division processes and development. Histone variant H2A.Z plays a critical role in early development, and *H2A.Z* RNAi results in defective chromosomal segregation (Faast *et al*., 2001; Rangasamy *et al*., 2004). Histone methylation is essential for meiosis, because it modulates the expressions of meiotic‐related genes (Wang *et al*., 2017). Additionally, histone acetylation status has a substantial impact on meiosis during both spermatogenesis and oogenesis (Wang *et al*., 2017).

We observed differential expression of a gene that encodes cyclin‐G‐associated kinase (GAK). GAK is required for proper mitotic progression because GAK knockdown was shown to cause cell cycle arrest at metaphase (Shimizu *et al*., 2009). An obvious cytological defect of CI is a delay in chromosome condensation in male pronuclei at prometaphase. Here, we observed differential expression of genes that encode DNA topoisomerase II. DNA topoisomerase II may play a role in chromosome condensation during mitosis (Wang, 2002). Furthermore, it is worth noting that two genes that encode the nuclear receptors ecdysone‐inducible protein E75 (E75) and ecdysone‐induced protein 78C (E78) were upregulated in Iws compared with Iw. In *Tribolium castaneum*, E75 regulates male accessory gland development and accessory gland protein production, whereas E78 is associated with sperm production and transfer to females (Xu *et al*., 2012). In *Drosophila*, females with low levels of *E78* expression experienced a notable decline in egg production and a low hatching rate compared with wild‐type individuals (Ables *et al*., 2015). These genes also regulate larval molting and development time (Bialecki *et al*., 2002).

In males, development time was found to be is inversely correlated with the strength of CI (Yamada *et al*., 2007). The spider mites we examined here that were co‐infected with *Wolbachia* and *Spiroplasma* produced more eggs, and the development time of doubly infected males was relatively short compared with that of males infected with only *Wolbachia*, and only the Iws line induced CI. Further research is needed to determine the effect of co‐infection on the expressions of the above genes in *T. truncatus*.

Some of the DEGs in the present study that are associated with the immune response were associated with co‐infection. These include the genes for the nuclear factor‐κB‐like transcription factor Relish, a Janus‐activated kinase/signal transducers and activators of transcription pathway ortholog of Dm stat92E, Toll‐like receptor Tollo and pelle. Moreover, a few genes related to autophagy and apoptosis also dramatically changed (Table S2). We could not confirm that co‐infection caused immune system activation, as we did not detect any antimicrobial peptide changes; therefore, further experiments are needed to determine if co‐infection activates the immune system. Identifying immune response diversity among different hosts and/or endosymbiont genotypes will also help clarify the interactions between endosymbionts and their hosts.

Furthermore, we observed numerous DEGs related to oxidoreductase activity due to co‐infection, such as the genes for dual oxidase, short‐chain dehydrogenase and peroxidase, which are important to maintain redox homeostasis.

## Conclusions

In summary, we demonstrated that symbiont infection could increase male mite hatchability and accelerate the development from egg to adult. We found that *Wolbachia* and *Spiroplasma* co‐infection affected transcripts that encode proteins involved in various biological processes in *T. truncatus*, such as digestion and detoxification, reproduction, the immune system and redox processes.

Our study provides novel gene‐based research on the effects of *Wolbachia* and *Spiroplasma* co‐infection on arthropod hosts, and helps elucidate the complex interactions between symbionts and arthropods.

## Disclosure

The authors declare no conflict of interest.

## Supporting information


**Table S1**. Primers used for quantitative real‐time polymerase chain reaction of differentially expressed genes.

**Table S2**. Genes that were differentially expressed in response to symbiont infection in *Tetranychus truncatus* males.
